# Model-Dependent EV Biomarker Signatures: From Culture Systems to Translational Perspectives

**DOI:** 10.3390/ijms27209325

**Published:** 2026-10-20

**Authors:** Giulia di Fazio, Giuseppina Poppa, Sandra D’Ascenzo, Vincenza Dolo, Simona Taverna, Ilaria Giusti

**Affiliations:** 1 Department of Life, Health and Environmental Sciences, University of L′Aquila, 67100 L’Aquila, Italy; giulia.difazio1@graduate.univaq.it (G.d.F.); giuseppina.poppa@univaq.it (G.P.); sandra.dascenzo@univaq.it (S.D.); vincenza.dolo@univaq.it (V.D.); 2 Institute of Translational Pharmacology (IFT), National Research Council (CNR), 90146 Palermo, Italy; simona.taverna@cnr.it

**Keywords:** extracellular vesicles, biomarkers, culture models, spheroids, cancer

## Abstract

Extracellular Vesicles (EVs) have emerged as key mediators of intercellular communication and, more recently, as promising biomarkers for disease diagnosis, prognosis, and therapeutic monitoring. In vitro cellular models are essential tools for investigating EV biogenesis, cargo composition, and biological functions. This review provides an overview of EV biology, including their classification, molecular content, biological functions and clinical relevance. It summarizes the main two-dimensional (2D) and three-dimensional (3D) culture systems currently used in EV research. The literature suggests that 3D models more closely recapitulate in vivo architecture, whereas conventional 2D cultures may introduce biological biases that affect experimental outcomes. While these aspects are presented from a simplified perspective to provide necessary background, the review primarily evaluates whether and how culture model selection influences EV production and features. In the final sections, we summarize and discuss evidence comparing EV yield, molecular cargo, functional properties, and biomarker profiles from 2D and 3D systems, and how they reflect in vivo settings. By examining these differences, we discuss their implications for interpreting EV-based studies and the importance of model choice in identifying and validating reliable EV biomarkers.

## 1. Extracellular Vesicles

Extracellular Vesicles (EVs) are membrane-enclosed, rounded particles released by virtually all cell types (humans, animals, plants and bacteria) and detectable in most biological fluids. They carry heterogeneous molecular cargo composed of proteins, lipids, and nucleic acids, such as mRNA, microRNA, and DNA, that reflects their cellular origin and state.

The most comprehensive definition of EVs was provided in the MISEV 2018 guidelines, which state that “ISEV endorses ‘extracellular vesicle’ (EV) as the generic term for particles naturally released from the cell that are delimited by a lipid bilayer and cannot replicate, i.e., do not contain a functional nucleus” [1].

Over time, the guidelines have been updated with MISEV2023 to reflect rapid advances in EVs, removing the term “naturally” from the definition, further refining the concept, and avoiding ambiguities associated with EVs generated or manipulated under experimental conditions [2].

The history of EV research can be traced back to the 1940s, when Chargaff and West investigated blood coagulation. In 1946, Chargaff and colleagues identified a particulate fraction with clotting activity that sedimented at 31,000× *g*, initially interpreted as small cellular fragments [3]. In 1967, Wolf used electron microscopy to observe small particles released by platelets and named them “platelet dust”. These particles, distinct from intact platelets, are now recognized as platelet-derived EVs [2,4]. In 1971, Crawford further characterized these structures as “microparticles,” demonstrating they contained lipids, ATP and contractile proteins, and suggesting a potential role in intercellular communication [5]. These pioneering studies established that circulating acellular particles were not merely cellular debris but biologically active structures, laying the foundation for the subsequent development of EV research [2].

### 1.1. EV Classification

Once these structures were identified, the first major challenge was their nomenclature. The general term “extracellular vesicles” was introduced and is currently used as a broad term for particles released into the extracellular space by virtually any cell type, regardless of differences in biogenesis and molecular composition.

The International Society for Extracellular Vesicles (ISEV) officially recommends this terminology (first in MISEV2018 and retained in the latest MISEV2023), which established standardised criteria for EV definition and classification [1,2].

A key objective is to unify the nomenclature, which continues to evolve. Historically, EVs were mainly classified by biogenesis. However, this classification is only valid when their cellular origin can be unequivocally demonstrated. It includes:

- Exosomes (EXOs, 40–120 nm), which originate from the endosomal pathway. This process begins with the internalization of plasma membrane components, forming early endosomes that then mature into late endosomes. Within late endosomes, the membrane undergoes multiple invaginations, forming multivesicular bodies (MVBs) containing intraluminal vesicles (ILVs). ILVs are eventually secreted as exosomes when MVBs fuse with the plasma membrane [6,7,8].
- Ectosomes or microvesicles (MVs, 50–1000 nm) arise from outward budding of the plasma membrane, driven by membrane remodelling and cytoskeletal reorganization [7,9].
- Apoptotic bodies (ABs, 500–2000 nm) originate from cells undergoing programmed cell death and are generated through plasma membrane blebbing and cellular fragmentation during the late stages of apoptosis [7,9].

Following the publication of the MISEV2018 guidelines, standardized recommendations for EV nomenclature and classification were established based on several characteristics. A first level of classification can be made according to EV physical properties and size, distinguishing small EVs (sEVs) from large EVs (lEVs). sEVs typically range from tens to several hundred nanometers in diameter, whereas lEVs can reach micrometer-scale dimensions [1].

Another useful feature for EV classification and identification is their biochemical composition, which can be characterized using specific molecular markers. These include membrane proteins, such as tetraspanins (e.g., EVs CD63+, CD81+, CD9+) as well as proteins associated with EV biogenesis and release that are associated with the ESCRT complex (e.g., EVs TSG101+, ALIX+) [1,2].

Further classification can be based on cellular origin. In this context, several EV subpopulations have been described, including microparticles (vesicles derived from circulating platelets or endothelial cells), tolerosomes (vesicles purified from the serum of antigen-fed mice), prostasomes (vesicles of prostatic origin), cardiosomes (vesicles secreted by cardiomyocytes), vexosomes (vesicles associated with adeno-associated viral vectors), dexosomes (dendritic cell-derived exosomes), matrix vesicles (found in bone, cartilage, and atherosclerotic plaques), oncosomes (tumor cell-derived vesicles), epididymosomes (vesicles originating from epididymal fluid), argosomes (vesicles derived from the basolateral membrane), synaptic vesicles (neuronal vesicles), and many other specialized EV subtypes [7,10].

EVs can also be described according to the physiological or pathological conditions under which they are produced. Accordingly, terms such as “hypoxic EVs” [11], “inflammatory EVs” [12], or the very common “tumor-derived EVs” [13,14] are used.

**Figure 1:**
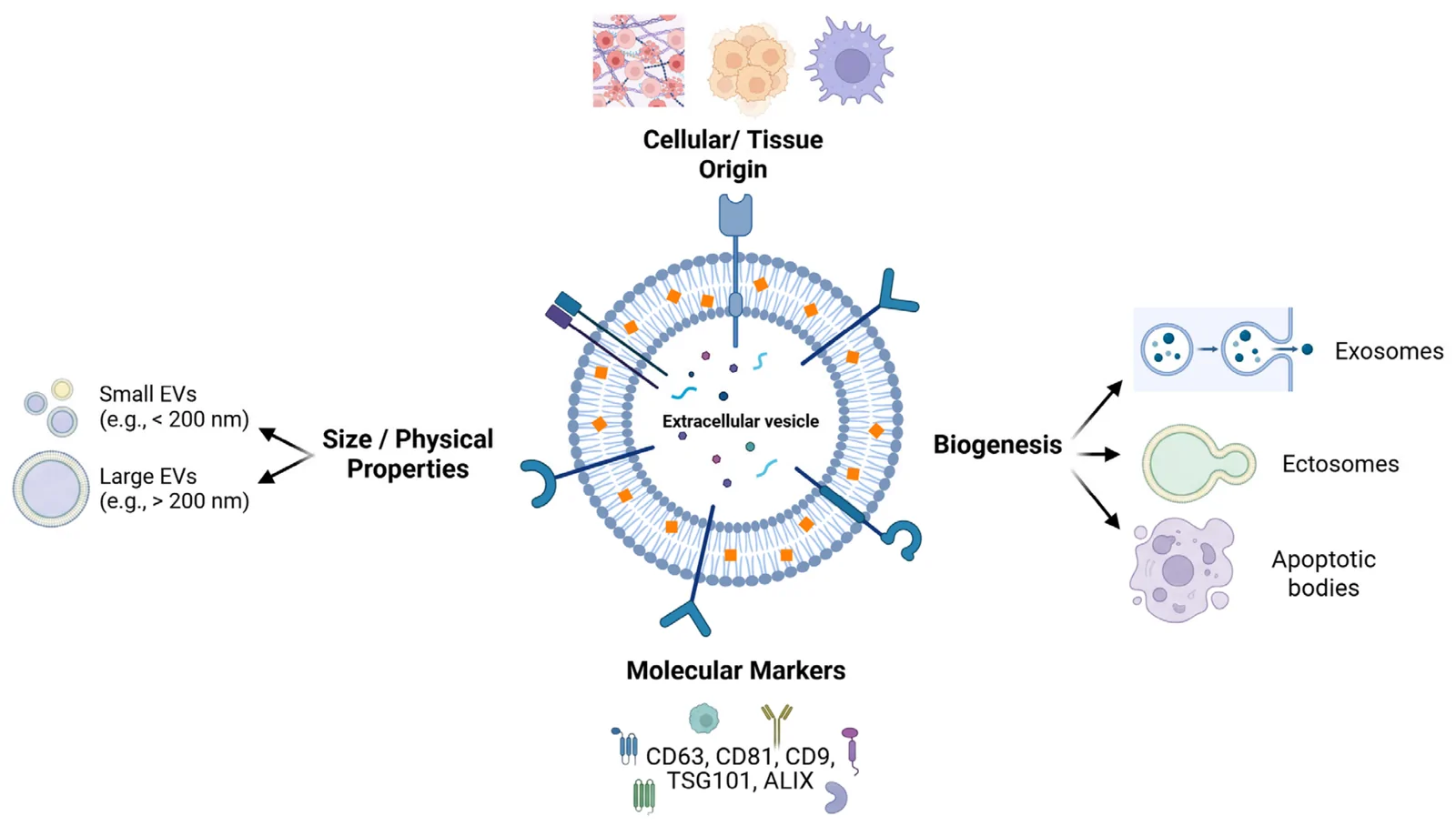
Schematic overview of extracellular vesicle (EV) classification. EVs can be categorized according to their biogenesis, physical properties, biochemical composition, cellular origin, and physiological or pathological state of the producing cells. Created in BioRender. Poppa, G. (2026) https://BioRender.com/0jzcxth.

The main criteria currently used for EV classification and nomenclature are schematically summarized in Figure 1.

However, the MISEV2023 update further emphasized using the generic term “extracellular vesicles” preferentially. The use of biogenesis-based terms, such as exosomes and ectosomes (microvesicles), should be discouraged or used with caution, unless the corresponding EV population has been specifically separated and rigorously characterized, with strong experimental evidence supporting its biogenesis pathway [2].

The terminology used throughout this review follows that adopted in the reference literature. Accordingly, when specific EV populations are discussed, the terminology used in the original cited studies has been retained.

Understanding how EVs are classified is essential to elucidate their functions, including their role in intercellular communication under both physiological and pathological conditions.

### 1.2. EV Composition

EVs carry biomolecules within their lumen and on their surface that reflect the parental cell’s cellular origin and physiological state, as well as the EV-biogenesis pathway. The molecular cargo varies across EV subpopulations and may exhibit both EV- and cell-type-specific characteristics. It includes: (i) proteins, which can provide insights into biological functions and their effects on cell-to-cell communication; (ii) lipids, which form a protective barrier for the vesicular cargo and serve as a recognition signal for target cells; (iii) nucleic acids, such as fragments of genomic or mitochondrial DNA as well as functional mRNAs, and also a great variety of non-coding RNAs (ncRNAs), such as microRNA (miRNA), circular-RNA (circ-RNA), and long non-coding-RNA (lncRNA) [15,16,17,18]. These molecules are extensively studied for their role in intercellular communication, as they can be transferred to recipient cells and modulate their biological and molecular functions [19]. Interestingly, although the type and quantity of RNA EVs reflect the molecular changes and biological events occurring in the cell of origin, their content still differs slightly from that of their parent cells [19,20].

EVs protect their cargo from degradation in the extracellular environment, allowing intact transport to target cells [21].

Because their diverse cargo, dedicated databases, such as ExoCarta and Vesiclepedia, have been developed and contain data from numerous EV studies [22,23].

Table 1 provides a schematic overview of the main EV components and their primary biological functions.

Alterations in EV composition can be detected in various biological fluids, making them useful as minimally invasive biomarkers that reflect the pathological conditions and hold considerable potential for clinical applications [28].

### 1.3. EV Functions

EVs can transfer information to neighbouring or distant target cells by delivering their cargo, inducing functional responses and contributing to both physiological and pathological changes. Under physiological conditions, EVs enable intercellular communication, ensuring cellular cooperation and proper tissue function [34,35]; conversely, under pathological conditions, EVs contribute to dysregulated cell-to-cell communication, thereby promoting disease onset and progression.

In physiological states, EVs contribute to cellular homeostasis by exporting unwanted or harmful components [36], participating in coagulation [37], and regulating stem cell maintenance [38], tissue regeneration [39], and cellular differentiation [40]. These functions highlight EVs’ fundamental role in maintaining biological balance. Moreover, their ability to transfer bioactive molecules and modulate recipient cell functions has stimulated interest in their therapeutic applications, particularly in regenerative medicine and immunotherapy [41].

However, despite their essential physiological roles in maintaining biological balance, EV-mediated communication can become detrimental under pathological conditions, as EVs may amplify or disseminate pathological signals throughout the organism. For example, EVs released by immune cells mediate intercellular communication and can exert bidirectional effects on biological responses, either enhancing or suppressing specific immune functions. In inflammatory settings, circulating EVs originate from multiple cell types, including platelets, endothelial cells, and leukocytes. They amplify inflammatory responses, thereby contributing to disease progression and associated complications. EVs in biological fluids can drive target cells toward pro- or anti-inflammatory phenotypes, thereby fine-tuning immune homeostasis [42].

EVs are particularly relevant in infectious diseases, where they may contribute to pathogen–host communication and to host cell damage by interfering with immune mechanisms and altering cellular responses [43,44].

EVs are involved in a wide range of tissue-level pathological processes. In organs such as the liver, lung, and cardiovascular system, they may contribute both to tissue injury and repair, influencing disease progression. In the central nervous system, EVs contribute to neuroinflammation and neurodegeneration by promoting microgliosis, astrogliosis, and synaptic dysfunction. In disorders such as multiple sclerosis, they are implicated in dysregulating regulatory T cells and maintaining chronic inflammatory states. In amyotrophic lateral sclerosis, they have been associated with the spread of toxic proteins and progressive neuronal degeneration [43].

Among the pathological conditions associated with EVs, cancer is one of the most extensively studied fields: indeed, EVs are central to communication between tumor and normal cells. Tumor-derived EVs often carry an altered molecular cargo, including oncoproteins, growth factors, cytokines, and microRNAs, that can influence recipient-cell behavior and promote disease progression [45].

Through the transfer of these bioactive molecules, they influence key cancer-related processes by promoting cell proliferation [43], angiogenesis [46], invasion [47], tumor progression [48], and metastasis [49], and actively contribute to tumor microenvironment (TME) modulation and pre-metastatic niche formation [50]. Overall, EVs emerge as crucial regulators of intercellular communication, capable of shaping both physiological homeostasis and disease progression [42,45,51].

## 2. Literature Search Strategy

Relevant literature was identified by searching PubMed, Scopus, and Web of Science using keyword combinations related to extracellular vesicles, biomarkers, 2D and 3D culture models, organoids, and liquid biopsy. Additional references were selected from the bibliographies of relevant articles. Given the narrative nature of this review, no formal systematic-review protocol was applied. The research was performed from April to August 2026.

## 3. In Vitro Cell Culture Models

In vitro cell cultures are essential models for studying EV biology, providing valuable insights into their biogenesis, molecular cargo, and biological functions.

Cell cultures allow the maintenance and propagation of cells outside their physiological environment in a controlled artificial setting that provides essential nutrients, growth factors, hormones, and gases, such as oxygen and carbon dioxide, while controlling physicochemical parameters including pH, temperature, and osmotic pressure. This approach provides a versatile research model for studying cellular physiology and biochemistry, pathological mechanisms, responses to drugs and toxic compounds, mutagenesis, carcinogenesis, drug action, and other aspects of cell biology. Most cells are anchorage-dependent and require attachment to a solid or semisolid substrate, whereas other cytotypes can be cultured in suspension within the medium [52].

Among cell culture models, primary cultures are derived directly from living tissue and may contain multiple cell populations from the tissue of origin; therefore, isolating the appropriate cell type is a crucial step. Their main limitations include complex isolation procedures and limited lifespan, although they retain genetic characteristics closer to in vivo conditions.

Alternatively, established cell lines, widely used in research and available from biological resource centers, provide well-characterized cellular models derived from numerous cell types and are extensively used in biomedical research [53].

Cell cultures allow consistent, reproducible results; for this reason, they are among the most widely used tools in cellular and molecular biology [53,54,55].

Depending on the mode of cell growth and organization, different culture models exist, each with specific advantages and limitations that can influence cellular behavior. Over time, these models have provided versatile platforms for investigating EV biology and identifying disease-associated biomarkers; in vitro EV research mainly employs two-dimensional (2D) and three-dimensional (3D) models.

### 3.1. 2D Models

For more than a century, 2D cell culture has been the most widely used model in biological research, driving advances in cellular and molecular biology, development of experimental disease models, new pharmacological therapies, and tissue engineering. This approach has enabled key scientific discoveries, including studies of antibiotic effects, vaccine development, generation of specialized cell lines, and production of induced pluripotent stem cells [53,56].

In 2D cultures, cells grow by adhering to a flat surface, typically glass or plastic, which provides mechanical support. Monolayer growth provides relatively uniform access to nutrients, oxygen, growth factors, and other molecules in the culture medium, as well as a stable temperature (37 °C), thereby promoting more homogeneous cell proliferation [57].

The number of cells obtainable from a 2D culture depends on the culture vessel’s available surface area. Once confluence is reached, cells are subcultured to avoid senescence or nutrient depletion in the culture medium.

The widespread use of 2D models stems from their ease of use and maintenance, low cost, high experimental reproducibility, the efficiency and simplicity with which functional studies and high-throughput assays can be performed, as well as the ease of collecting conditioned medium for EV isolation [56].

Despite their extensive use, 2D cultures present several limitations. Because growth occurs on a flat surface, these models often do not adequately recreate the 3D morphology of cells or the complexity of the in vivo tissue microenvironment, including 3D cell-to-cell interactions and interactions with the extracellular matrix (ECM), which characterize the physiological in vivo environment. Moreover, uniform nutrient and oxygen availability differs from physiological tissue conditions, where these components are distributed according to spatial gradients [57].

Thus, 2D cultures cannot faithfully reproduce the 3D architecture of tissues and may alter cell morphology, polarity, protein and gene expression, and various biological processes, including polarization, proliferation, differentiation, migration, cell signaling, and responses to stimuli [53,56].

Another limitation is that 2D cultures are often composed of a single cell type, allowing the study of only one cell population and failing to reproduce the heterogeneity of the tissue microenvironment [53].

To overcome some limitations of conventional 2D cultures, researchers have developed several techniques, including micro-patterned substrates such as microwells [58], modulation of substrate stiffness using materials with different mechanical properties, and systems incorporating extracellular matrix components, such as sandwich cultures. These approaches allow reproduction of certain aspects of the cellular microenvironment and influence processes such as cell adhesion, morphology, and function [56].

However, 2D cultures still cannot fully reproduce the structural, functional, and physiological complexity of tissues in vivo [56].

**Figure 2:**
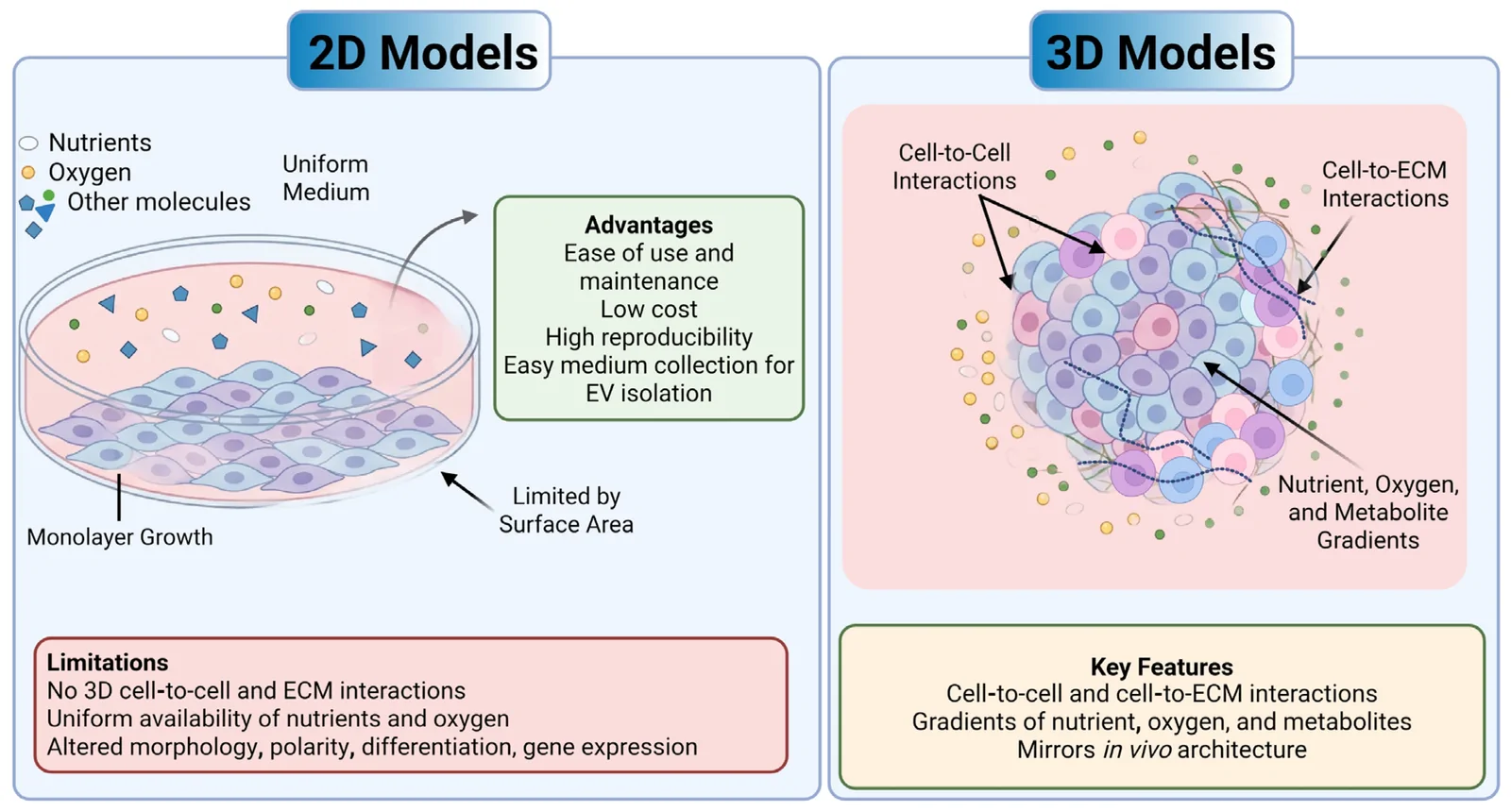
Schematic comparison between 2D and 3D culture models. In 2D cultures, cells grow as a monolayer in a uniform medium, providing ease of use, low cost, and high reproducibility, but lacking physiologically relevant cell-to-cell and cell-to-ECM interactions. In contrast, 3D cultures establish a spatially organized architecture that promotes cell-to-cell and cell-to-ECM interactions and generates nutrient, oxygen, and metabolite gradients that more closely resemble the in vivo microenvironment and tissue organization. Created in BioRender. Giusti, I. (2026) https://BioRender.com/69oolfm.

For these reasons, interest in 3D culture models has increased in recent years. These models were developed to more faithfully recreate the cellular microenvironment than traditional 2D cultures (Figure 2).

### 3.2. 3D Models

In these models, cells grow within a 3D structure that promotes cell-to-cell and cell-to-ECM interactions, essential for maintaining cellular functions and tissue organization. In addition, the 3D arrangement of cells creates nutrient, oxygen, and metabolite gradients that more closely mirror in vivo conditions [56,59].

These systems bridge traditional 2D cell cultures and animal models, offering an economical, scalable, and ethical alternative for preclinical research [60].

Designing a 3D model requires evaluating several parameters, including cell type, tissue origin, presence of ECM-mimicking components, growth factors, and culture conditions. For this reason, numerous systems have been developed, each one with specific characteristics and different applications.

3D culture models are generally classified into two main categories: scaffold-free systems and scaffold-based systems.

In scaffold-free models, cells spontaneously self-aggregate through cell-to-cell interactions, forming suspended 3D structures. In contrast, scaffold-based models use natural or synthetic supports that provide structural support and partially recreate ECM characteristics [54,56,60]. These models can be further combined with static and dynamic systems: static models keep cells immersed in the culture medium without continuous flow, whereas dynamic models continuously flow the culture medium through microchannels, creating a moving environment [61].

An example of scaffold-free 3D culture are spheroids, i.e., cellular aggregates that grow in suspension and are composed of established cell lines; they are easy to generate because they do not require scaffolds [62].

**Figure 3:**
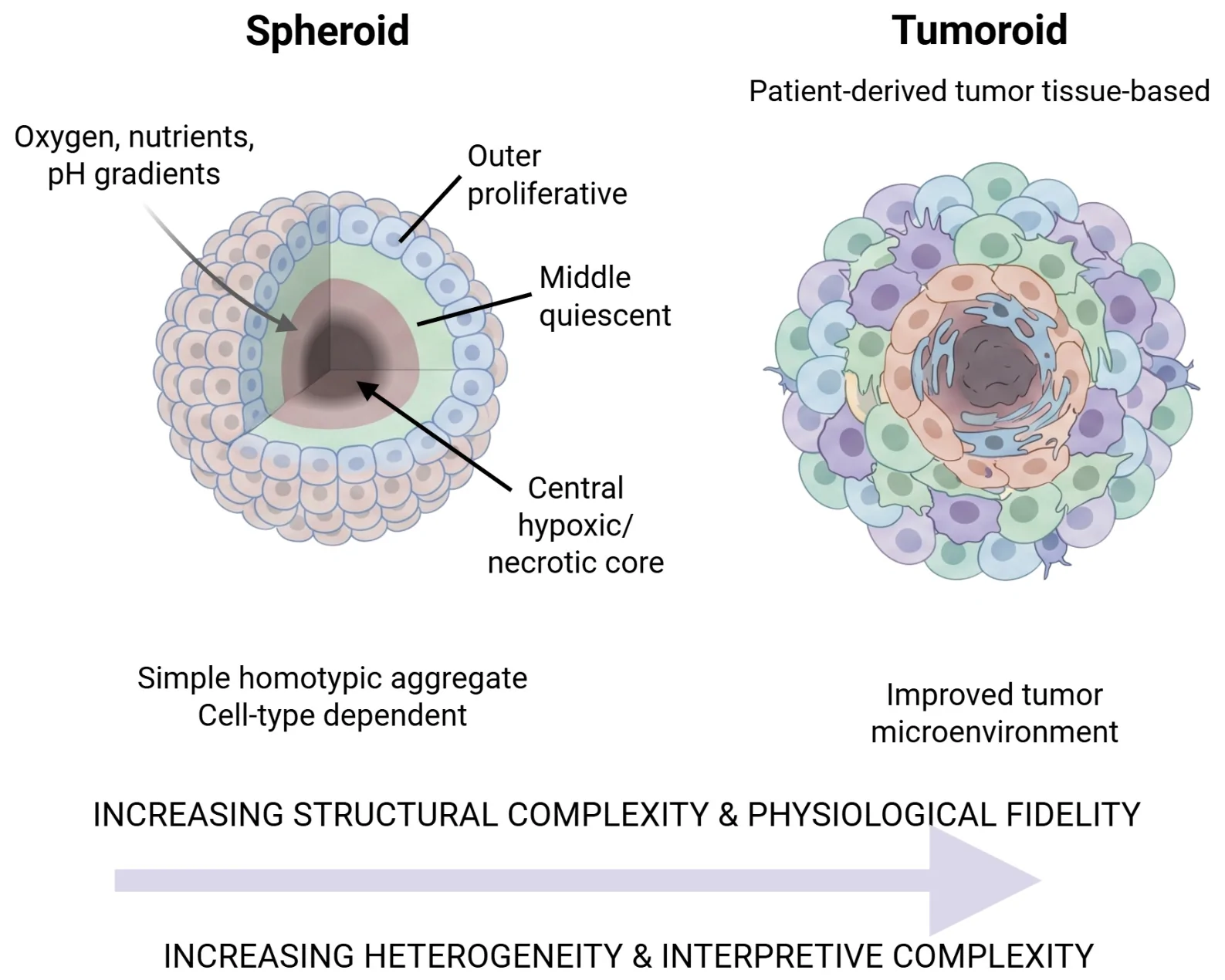
Overview of major 3D culture models in cancer. Spheroids (on the **left**) are cell aggregates characterized by oxygen, nutrient, and pH gradients that typically show an outer proliferative layer, an intermediate quiescent region, and a central hypoxic or necrotic core. Tumoroids (on the **right**), generated from patient-derived tumor tissues, better reproduce tumor heterogeneity and microenvironmental features. From spheroids to tumoroids, structural complexity, physiological relevance, and cellular heterogeneity progressively increase, together with the complexity of data interpretation. Created in BioRender. Poppa, G. (2026) https://BioRender.com/8hmjj20.

Spheroid models are most commonly used to study cancer. Tumor cells form spherical structures with organized architecture and variable size, characterized by a necrotic core and peripheral cellular layers. Depending on spheroid size, three distinct regions can be identified: an outer zone of proliferating cells, an intermediate region of quiescent cells, and a central necrotic, hypoxic area. This organization creates oxygen, nutrient, and pH gradients, with availability progressively decreasing from the periphery to the center, partially recapitulating the complexity typical of the tumor microenvironment [63] (Figure 3, left panel).

Tumor spheroids can be broadly classified as homotypic or heterotypic. Homotypic spheroids, also called monocellular or homospheroids, develop from a single tumor cell type and are commonly used to study cellular behavior, physiological characteristics, and cell morphology in a controlled environment [64]. In contrast, heterotypic spheroids, also known as mixed or heterospheroids, comprise multiple cell populations and more closely resemble tumor cellular heterogeneity in vivo [65]. Because tumors are complex and include different cellular (malignant and stromal) and non-cellular components, monoculture spheroids cannot fully recapitulate tumor complexity. To address this limitation, researchers have developed more sophisticated heterotypic models with varying levels of complexity by co-culturing tumor cells with stromal and other supporting cell types. These systems provide a more physiologically relevant platform for studying tumor biology and a greater clinical relevance for in vitro therapeutic screening [64].

Despite these advantages, spheroids remain relatively simple, homogeneous and standardized models, being based on cell lines; furthermore, aggregate formation and compactness depend on the cell type used, limiting this model to selected cell lines [66,67].

To better reflect the heterogeneity typical of patient-derived tumors, tumoroids have emerged as a further evolution of 3D tumor models (Figure 3, right panel).

The term “tumoroid” refers to a heterogeneous group of patient-derived tumors, including cultures established from primary tumor cells and those obtained directly from ex vivo tumor tissue. Native tumoroid models are derived from surgical or biopsy specimens that undergo minimal manipulation, preserving native tumor architecture, its cellular composition and key TME characteristics. These models can sustain tumor and immune cell populations over time, providing a physiologically relevant platform for studying tumor–immune system interactions and evaluating treatment responses [68].

However, nomenclature for patient-derived 3D tumor models remains poorly standardized. Although some studies use “tumoroid” and “patient-derived tumor organoids” interchangeably [68,69], considerable ambiguity remains in defining and classifying tumoroids, tumor organoids, and related 3D culture systems [70]. This likely reflects, at least in part, the ongoing evolution of these models, which are continually refined through different procedural approaches.

Another scaffold-free model, mostly used to study non-tumor diseases, is represented by organoids. Compared with in vitro 2D systems, organoids are more complex because they include different interacting cell types that can self-assemble. While this complexity can make results harder to interpret and attribute to individual cell populations, it also enables organoids to better reproduce the organization and characteristics of in vivo tissues. Furthermore, because they are often derived from primary cells, organoids retain some features of their tissue of origin while also reflecting the biological variability observed among individuals [68,71]. However, it should be noted that organoids are most established in scaffold-based matrices (e.g., Matrigel or natural/synthetic hydrogels), which mimic key features of the ECM and promote 3D tissue organization [72].

**Figure 4:**
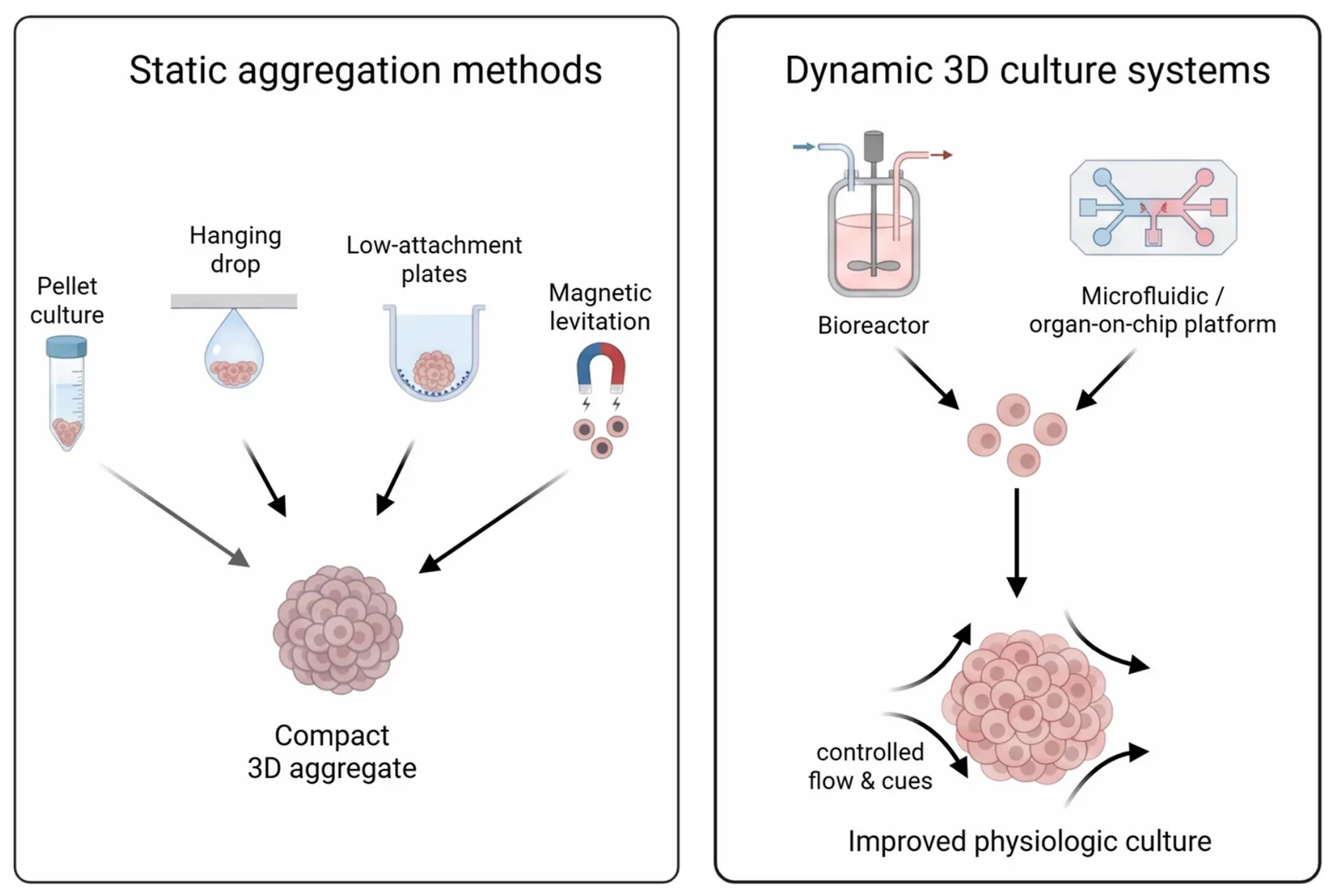
Main strategies for the generation of three-dimensional cell culture models. Static aggregation methods, including pellet culture, hanging-drop culture, low-attachment plates, and magnetic levitation, promote the self-assembly of cells into compact spheroids/cell aggregates. Dynamic culture systems, such as bioreactors and microfluidic/organ-on-chip platforms, expose cells to controlled fluidic and mechanical stimuli, improving nutrient transport and enhancing the physiological relevance of in vitro models. Created in BioRender. Giusti, I. (2026) https://BioRender.com/9bdqt2n.

Several techniques can generate these 3D models, often using static culture systems (Figure 4).

The main approaches include:

- Pellet Culture, in which cells are centrifuged to promote cell–cell interactions and aggregate formation; the resulting pellet is then resuspended in culture medium, allowing spheroid growth [73].
- Hanging drop, a technique that consists of placing droplets containing a cell suspension on the inner surface of a culture plate lid, which is subsequently inverted. Alternatively, cells can be cultured in specialized plates designed for hanging-drop culture. Under these conditions, the combined action of gravity and surface tension promotes spontaneous cell aggregation at the bottom of the drop [74].
- Low-attachment or Ultra-Low Attachment plates, in which the cell suspension is seeded into wells characterized by anti-adhesive surfaces. The absence of substrate adhesion and the specific geometry of the wells promote cell-to-cell interactions and the spontaneous formation of three-dimensional aggregates [54,75].
- Magnetic levitation, where the cells are initially magnetized through the incorporation of magnetic microspheres and subsequently seeded into low-adhesion plates. Applying a magnetic field promotes rapid cell aggregation and the formation of compact 3D structures [76].

Scaffold-based 3D cell cultures use supporting structures that provide a 3D environment promoting cell-to-cell and cell-to-ECM interactions. Cells can be seeded onto a preformed scaffold or within liquid matrices that subsequently solidify or polymerize [77].

Among scaffold types, hydrogels are the most widely used; they consist of 3D networks of hydrophilic polymers that can absorb large amounts of water. Hydrogels are generally classified as static systems and are widely used because they allow easy cell embedding and because their mechanical and physical properties can be modulated. Furthermore, their high water content enables them to reproduce certain extracellular matrix characteristics and facilitates diffusion of nutrients, metabolites, cytokines, and growth factors.

Hydrogels can be formed from natural, synthetic, or hybrid materials. Natural materials include collagen, gelatin, hyaluronic acid, alginate, chitosan, and basement membrane derivatives such as Matrigel^®^ and Cultrex^®^ [77,78].

Among natural hydrogels, collagen is one of the most widely used materials because it is a major ECM component. Varying concentration and preparation conditions can modulate the mechanical properties, architecture, and degradability of collagen gels. Another commonly used material is Matrigel^®^, a mixture of basement membrane proteins rich in laminin, type IV collagen, proteoglycans, and growth factors. Due to its composition, Matrigel^®^ promotes cellular differentiation and the formation of complex 3D structures. However, its animal origin results in batch-to-batch variability and a composition that is not fully defined [57,77,79,80].

Among synthetic materials, polyethylene glycol, polyvinyl alcohol, poly(lactide-co-glycolide), and polycaprolactone are commonly used. The choice of biomaterial depends on its intended application and the required biochemical, structural, and mechanical properties [77].

Despite their numerous advantages, hydrogels present some limitations. For synthetic scaffolds, certain physicochemical characteristics, such as hydrophobicity, may limit cell adhesion and proliferation, often necessitating surface treatments to improve biocompatibility. In addition, in many cases the polymeric network of hydrogels contains pores of limited size, which may hinder cell migration and matrix colonization after scaffold formation. To overcome these limitations, hydrolytically or enzymatically degradable materials have been developed, that allow the progressive formation of new spaces within the scaffold during culture [56].

Another category includes preformed polymeric scaffolds, which allow control over biochemical composition, elasticity, porosity, and 3D architecture. They can be produced through various techniques, such as 3D printing [81], stereolithography [82], freeze-drying, polymer phase separation, gas foaming [83], and porogenic materials [84]. Many fabrication processes involve conditions incompatible with cell culture and cell survival; consequently, cells are generally introduced into the scaffold after its fabrication. A limitation of this approach is that cell distribution within the structure is not completely uniform [56].

To improve cell proliferation, polymeric scaffolds assembled from building blocks have been developed, allowing more precise control over porosity, mechanical properties, and extracellular matrix characteristics. These systems promote nutrient diffusion, cell proliferation, and new extracellular matrix deposition [60].

Scaffold architecture plays a key role in regulating cellular behavior. To better mimic the native tissue environment, scaffold design can be engineered by adjusting parameters such as porosity, pore size and geometry, pore interconnectivity, surface topography, and mechanical properties. These characteristics regulate nutrient and oxygen transport, waste removal, cell adhesion, and proliferation and differentiation processes [55,56,61].

Three-dimensional models can also be combined with bioreactors, making culture models dynamic (Figure 3). These systems allow the regulation and monitoring of parameters such as temperature, pH, oxygenation, nutrient distribution, and metabolite removal. Common types include rotating-wall bioreactors such as spinner flasks and rotating-wall vessels, perfusion systems, hollow-fiber bioreactors, and systems that apply controlled mechanical stimulation. With these features, dynamic models can recreate conditions closer to those found in vivo [55,60].

Microfluidic culture systems are another type of dynamic model. These devices maintain cells in a dynamic environment through controlled culture-medium flow, generating shear forces that influence cellular behavior. Organ-on-chip technologies have been derived from these systems; these platforms combine different cell types and three-dimensional microenvironments to reproduce some structural and functional characteristics of organs. Because they allow control of physical and biological parameters, these systems are widely used to study pathological mechanisms, drug screening, and toxicity testing. However, their implementation is complex, requires a limited number of cells, and their ability to predict in vivo responses more accurately than other models remains under evaluation [84].

3D models have also expanded in EV research, as numerous studies have highlighted significant differences in the quantity, composition, and functional properties of EVs isolated from 3D cultures compared to those obtained from 2D models.

## 4. Culture Models Affect EV Yield, Molecular and Functional Signatures

Numerous studies show that the cell culture model significantly influences EV production, molecular composition, and functional properties. This review is not intended to be systematic; instead, this section presents non-exhaustive examples of how 2D and 3D culture systems can modulate EV signatures across different cellular and pathological contexts. We primarily focused on original studies directly comparing EVs produced under 2D and 3D culture conditions using the same cellular model. In addition, selected review articles were included to provide broader context. These studies are summarized in Table 2, which reports quantitative, molecular, and/or functional EV outcomes.

The impact of culture model on EVs can be considered from three main perspectives: (i) quantitative changes in EV yield, (ii) qualitative differences in EV molecular composition, and (iii) functional modifications of EV biological activity.

(i) From a quantitative perspective, direct comparisons between 2D and 3D culture systems have reported heterogeneous effects on EV yield, with 3D cultures generally producing higher amounts of EVs than 2D models, as reviewed in [54]. Several studies have reported higher EV production in 3D cultures [85,86,87,88], with some exceptions where no statistically significant difference has been found between 2D and 3D conditions [89,90]. The literature also emphasizes the advantage of 3D systems, including bioreactor-based approaches, for achieving higher EV yields through scalable, space- and cost-efficient culture strategies, which is critical for the future clinical translation of EV-based therapies [91]. In the context of stem cell-derived EVs, culture conditions have also been reported to influence EV production, with 3D culture systems providing a more physiologically relevant environment by enhancing cell-to-cell and cell-to-ECM interactions [92]. By comparing EVs released by glioblastoma cells cultured in 2D and 3D organoid models, an increased EV concentration was observed in 3D cultures compared with conventional 2D cultures [85]. Another study on glioblastoma showed that spheroids tended to release more EVs than 2D cultures; although the difference was not statistically significant, it also highlighted a shift in size distribution, with EVs from 3D cultures significantly smaller than those from 2D cultures [89].

A comparison of EVs released by gastric cancer cell lines cultured under 2D and 3D conditions showed that 3D cultures produced a significantly higher number of EVs per cell. In addition, EVs derived from 3D cultures were smaller, with mean sizes ranging from 85 to 135 nm compared with 100–180 nm for EVs derived from 2D cultures [86].

**Table 2:** Impact of 2D and 3D culture conditions on extracellular vesicle production, molecular composition, and biological activity.

Cell Type	3D Culture System	EV Isolation and Normalization	EV Cargo Changes	Effect on EV Yield/Size	**Functional Changes**	**Ref.**
Glioblastoma cells from patients	Organoids	Precipitation and immunoaffinity NTA-derived EV concentrations were normalized using a culture-specific index (3D cell counts unavailable) and corrected for dilution.	9 and 3 miRNAs are down- and upregulated, respectively, in 3D organoids	Higher EV concentration in 3D	GBM progression and immunosuppressive environment	[85]
Glioblastoma cell line U87MG	Spheroids	SEC EV particle number normalized to 8 × 10^6^ cells	Distinct proteomic profiles; 2D EVs enriched in signaling, ECM interaction, and stress-response proteins; 3D EVs enriched in EV trafficking and metabolic adaptation; different TMZ response	3D vs. 2D EVs: significantly smaller, but not significantly more in yield	Culture architecture affects EV signatures and response to TMZ	[89]
Gastric cancer cell lines MKN74 and MKN45	Spheroids	DC and UC Total EV number normalized to the total number of cells	microRNA upregulation, combined with the downregulation of the target proteins in 3D EVs	3D vs. 2D EVs: significantly increased in yield, represented by a smaller population	Culture architecture affects EV biogenesis and cargo	[86]
Human healthy women umbilical cord-derived MSCs	Bioreactor-based 3D culture	DC and UC EV yield compared using the same total volume of conditioned medium collected from each system (600 mL); not normalized per cell number	Not specified in this section	Approximately 8.21-fold higher EV production in 3D	Enhanced cardioprotective effects: reduced cardiomyocyte apoptosis, promoted angiogenesis, regulated macrophage transitions	[87]
Human choriocarcinoma JAr cells	Spheroids	SEC Same initial cell number (1 × 10^6^ cells) and same collection period; EV concentration measured by NTA	Some cargo protein (e.g., ANXA2, SLC3A2, RAC1) enriched in 3D vs. 2D EVs	Higher EV concentration in 3D vs. 2d EVs; no significant differences in size	Cell–cell adhesion, cell migration, ECM receptor interaction, regulation of actin-cytoskeleton and Ras signalling; differential modulation of MFGE8 secretion in receptive endometrial cells	[88]
Bone marrow-derived MSCs from healthy patients	Spheroids	DC and UC Total crude EV protein yield (BCA); EV particle yield (SP-IRIS); 2D and 3D cultures maintained at the same cell density (5 × 10^4^ cells/mL) and culture duration	Different protein cargo between 2D- and 3D-derived EVs	No difference in size and yield detected	Immunomodulatory activity and stronger immunosuppressive, phagocytic, and antifibrotic effects	[90]

Another study using human umbilical cord-derived mesenchymal stem cells (MSCs) found that a bioreactor-based 3D culture system produced approximately 8.21-fold more EVs than conventional 2D culture; EV yield was assessed by NTA, comparing EV production from an equal starting volume of conditioned medium (600 mL) collected from the 3D and 2D systems [87]. A similar comparison in JAr choriocarcinoma cells also reported a higher EV particle yield under 3D conditions [88].

It is to be noted, however, that some studies reported no significant difference in EV yield, as was observed between 2D and 3D cultures of bone marrow-derived MSCs [90].

(ii) Regarding qualitative differences, several studies have demonstrated that the culture model influences the molecular cargo and biomolecular profile of EVs. It has been reported by some studies that while EV morphology is often similar in both 2D and 3D models, EVs from 3D cultures exhibit mRNA and miRNA profiles more closely aligned with those observed in vivo [54]. Several papers, many of which are comprehensively reviewed in [92], have reported that differences in culture architecture and microenvironment between 2D and 3D systems can influence cell behaviour and the molecular characteristics of secreted EVs. 3D culture systems, being more similar in architecture to in vivo conditions, appear to alter molecular cargo composition and improve biological activity, including their ability to promote angiogenesis and wound healing, as well as their anti-inflammatory, anti-apoptotic, and anti-fibrotic effects.

Another study comparing EV release from glioblastoma cells cultured in 2D versus 3D found differences in EV molecular content, with 3D cultures showing a different miRNA expression profile. The authors conclude that the culture model used can substantially influence EV characteristics and suggest that 3D glioblastoma models may be the most suitable for clinical biomarker studies and for investigating new therapeutic strategies [85].

Another study found distinct proteomic profiles in EVs derived from 2D and 3D glioblastoma cultures under basal conditions. EVs from 2D cultures were enriched in proteins involved in signal transduction, extracellular matrix interactions, and stress responses. In contrast, EVs from 3D spheroids were enriched for proteins associated with vesicle trafficking and metabolic adaptation. Temozolomide treatment also revealed differences: 2D-derived EVs showed more pronounced proteomic remodelling, with enrichment of proteins associated with DNA damage response, translational regulation, and signalling; in contrast, EVs from 3D spheroids showed a more limited proteomic response to temozolomide treatment [89].

Further studies have confirmed the influence of the culture model on the biomolecular profile of EVs: for example, EVs generated from 3D cultures of human choriocarcinoma cell line JAr displayed a different protein profile compared with those generated from 2D cultures, including proteins involved in cell–cell adhesion, cell migration, and interaction with the ECM [88]. Similarly, a study of bone marrow-derived mesenchymal stem cells revealed, by proteomic analysis, a different protein cargo between 2D- and 3D-derived EVs, supporting the idea that the culture microenvironment influences EV composition and biological activity [90].

One study compared the molecular characteristics of EVs released by gastric cancer cell lines. Proteomic analysis revealed distinct protein profiles between EVs derived from 2D and 3D cultures, with 17 of the 20 most significantly different proteins showing lower expression in 3D-derived EVs. Although the overall miRNA profiles were largely similar between the two culture conditions, some miRNAs were detected exclusively in EVs derived from either 2D or 3D cultures [86].

(iii) From a functional perspective, several studies have shown that the differences in EV composition induced by the culture model can influence, as might be expected, their biological activity. Generally, as comprehensively reviewed in [91], 3D models used to investigate the therapeutic effects of EVs have, in several cases, revealed stronger anti-inflammatory effects. EVs derived from 3D-cultured MSCs improve therapeutic effects [93], osteogenic [94], and wound-healing [95] properties compared with those derived from 2D cultures.

Variations in protein composition in 2D- vs. 3D-derived EVs were also linked to changes in functional activity, affecting cell communication in an embryo implantation model: 2D- and 3D-derived EVs differentially modulated MFGE8 secretion, a molecule critical for embryo implantation, in a receptive endometrial cell line [88]. Overall, these results indicate that the culture model influences EVs’ molecular and functional characteristics, highlighting the importance of standardizing experimental models to improve clinical translation.

However, it should be noted that EVs derived from 3D models are not always functionally superior. In a study on lung fibrosis investigating how MSC growth as 2D or 3D spheroid cultures affects EV production, bioactivity, and molecular composition, EVs from bone marrow MSCs cultured in 2D showed up to 5 times greater immunomodulatory capacity than those cultured in 3D. Furthermore, functional assays suggested that 2D-derived EVs also better promoted immunosuppression and enhanced phagocytic and antifibrotic activity. These differences were corroborated in vivo in a murine pulmonary fibrosis model, where administration of EVs from both 2D and 3D cultures showed that 2D-derived EVs reduced collagen deposition, myofibroblast differentiation, and leukocyte infiltration, but 3D-derived EVs did not show the same beneficial effects [90].

Functionally, a study of EVs derived from 3D cultures of human umbilical cord-derived MSCs showed enhanced cardioprotective effects compared with those derived from 2D cultures, repressing cardiomyocyte apoptosis, facilitating angiogenesis, regulating macrophage subpopulation transitions after myocardial infarction, and eventually improving cardiac function in a rat model of acute myocardial infarction [87].

Beyond the biological differences between 2D and 3D culture systems, several methodological variables may also contribute to the heterogeneity of EV preparations and should therefore be considered when interpreting and comparing experimental results.

Cell culture conditions represent an important source of variability in EV studies, as factors such as medium composition, the use of serum containing EVs or EV-depleted media, cell confluency, hypoxia, passage number and culture platform can influence EV release, composition and biological activity [96,97].

The choice of isolation workflow is another parameter that can affect EV recovery, purity, and molecular composition, with different approaches showing variable efficiency in separating EVs from contaminating extracellular components. Different isolation methods enrich distinct EV populations according to their size, density, biophysical characteristics, or surface-marker expression. Consequently, the choice of isolation strategy can further influence the composition and purity of the recovered EV population.

Moreover, 3D culture platforms add complexity due to presence of biomaterials and extracellular matrices. Indeed, interactions between EVs and matrix components may affect EV recovery, as a fraction of secreted EVs may remain associated with hydrogel- or matrix-based scaffolds rather than being released into the conditioned medium. Although such retention can be exploited for therapeutic purposes to achieve localized and sustained EV delivery, it may hinder efficient isolation of EVs from hydrogel-embedded cultures, affecting downstream quantitative and qualitative analyses. In addition, when 3D cultures are used, the establishment of oxygen and nutrient gradients can also promote apoptosis or necrosis in the inner core, potentially contributing additional vesicular and non-vesicular particles to the collected samples [54,91,98].

Consequently, accurate reporting of culture conditions, sample processing, EV isolation procedures, and characterization methods is essential to ensure reproducibility and enable meaningful comparisons across studies, in line with MISEV2023 recommendations [2].

In conclusion, numerous studies, including those discussed, show that culture model conditions significantly affect the biomolecular profile and functional properties of EVs. Both 2D and 3D models have specific advantages and limitations that should be considered based on experimental objectives and intended applications, as neither model can be considered universally superior. Standardizing EV isolation, characterization, and production protocols is also crucial to ensure reproducibility and support clinical translation.

## 5. EVs as Biomarkers and the Importance of the Appropriate Preclinical Model

EVs’ ability to mediate intercellular communication, along with molecular cargo that matches parental cell features, has made them promising candidates for disease biomarker discovery [99].

Once released from their cell of origin, whether normal or tumor, EVs can reach several biological fluids, including blood, saliva, urine, pleural effusion, cerebrospinal fluid, ascitic fluid, amniotic fluid, and breast milk, where their presence makes them particularly attractive targets for liquid biopsy-based approaches [100,101]. Although EV-based liquid biopsy is increasingly applied to both neoplastic and non-neoplastic diseases, this review focuses primarily, but not exclusively, on cancer.

EVs as biomarkers offer several clinically relevant advantages. Their isolation from biofluids is generally performed using minimally invasive procedures, making it more patient-friendly and better tolerated, while providing comprehensive information on disease status in a minimally invasive manner [102].

Given the high heterogeneity of tumors, identifying a single diagnostic biomarker is often challenging; in this context, EVs are particularly promising because they simultaneously carry multiple biologically relevant molecules, including RNA, DNA, and oncogenic proteins [103]. In addition, the vesicular membrane protects the molecular cargo from degradation, providing greater stability in stored patient samples than freely circulating biomolecules [104]. Another key advantage is their potential for high diagnostic specificity and sensitivity, as studies evaluating EV-associated biomarkers in different clinical settings have shown. For example, an exosome-associated SORL1 detection platform for colorectal cancer achieved an area under the curve of 0.99, compared with 0.71 for the conventional biomarker carcinoembryonic antigen (CEA), while showing comparable diagnostic performance in both early-stage colorectal cancer and CEA-negative patients [105]. These findings highlight the potential of EV-associated biomarkers to improve diagnostic accuracy and support earlier detection and more personalized clinical management [105,106]. These features give EVs potential for applications such as early disease detection and dynamic monitoring of disease progression and therapeutic response, allowing tracking of disease evolution over time and potentially supporting more targeted and personalized treatment strategies [107].

Among the classes of EVs identified to date, research has historically focused primarily on sEVs (often referred to as exosomes in the scientific literature), and diagnostics research is expanding with promising results [108]. Despite this focus on sEVs, however, new evidence suggests that lEV populations may also represent valuable sources of clinically relevant biomarkers [109,110,111,112,113].

Table 3 summarizes, albeit necessarily non-exhaustively, studies reporting increased/decreased expression of EV-associated molecules in blood, urine (the most commonly used biological samples), or other fluids as potential biomarkers for malignant and non-malignant diseases.

Despite the large number of EV-associated biomarkers proposed for cancer diagnosis and patient stratification, few EV-based liquid biopsy assays have reached clinical implementation.

The most important examples of clinical translation are represented by the ExoDx™ Lung and ExoDx Prostate IntelliScore (EPI) platforms. ExoDx™ Lung exploits the so-called ExoNA platform, which combines exosomal RNA/DNA and circulating cell-free DNA to improve mutation detection in plasma samples. The assay enables the identification of clinically relevant EGFR alterations, including activating mutations (L858R and exon 19 deletions) as well as the resistance-associated T790M mutation [153,154].

On the other hand, EPI is a non-invasive urine test intended for men aged ≥ 50 years with prostate-specific antigen (PSA) levels between 2 and 10 ng/mL who are being considered for an initial prostate biopsy. After urinary exosome isolation, the expression of three RNA biomarkers, ERG, PCA3, and SPDEF, is measured and integrated into a risk score ranging from 0 to 100 [155,156,157].

Despite growing interest in the clinical use of EVs as diagnostic and prognostic biomarkers, several technical challenges still hinder their translation into routine clinical practice. These challenges primarily concern rapidly, accurately, and reproducibly isolating EVs while minimising co-isolation of non-vesicular contaminants and other particles that may interfere with downstream analyses [158]. Among available approaches, ultracentrifugation is one of the most widely used methods for EV isolation because of its simplicity and established protocol; however, it remains time-consuming and expensive, and recovery and purity depend on the biological matrix used [21,159].

For example, in biological fluids such as plasma, the presence of abundant non-vesicular particles, including lipoproteins and protein complexes, enables their co-isolation with EVs during the separation process because of their physicochemical properties; thus, it represents a major challenge for downstream molecular analysis and affects the identification and validation of candidate biomarkers [160]. Alternative approaches to ultracentrifugation techniques, including polymer-based precipitation, ultrafiltration, size-exclusion chromatography, immunoaffinity methods, microfluidic technologies, and others, have been proposed as faster, more clinically applicable strategies [161,162,163]. However, each approach has advantages and limitations related to recovery, purity, throughput, and selectivity for specific EV populations [159,162,164]. For instance, polymer-based precipitation approach offers high recovery and simple, rapid processing; however, the resulting sample has reduced purity because non-vesicular contaminants co-precipitate, whereas size-exclusion chromatography generally yields higher purity but may require pre-concentration and provide lower recovery [152]. On the other hand, immunoaffinity-based and microfluidic methods can improve selective enrichment of specific EV subpopulations and enable rapid processing, respectively; however, their application may be limited by dependence on specific surface markers, low sample throughput, clogging risks, and the lack of standardized protocols [165,166]. Table 4 summarizes the main advantages and disadvantages of the principal EV isolation methods.

The isolation strategy itself may influence the resulting EV preparation; differences in pre-analytical procedures, EV characterization, and analytical platforms can also affect reproducibility and hinder comparisons between studies [178]. For this reason, the latest ISEV guidelines emphasize rigorous, standardized approaches to EV separation and characterization, including appropriate assessment of non-EV components and the use of complementary analytical methods to facilitate translating EV-based biomarkers into clinical practice.

In a clinical translational perspective, the isolation issue is not the only one to be considered. Indeed, beyond EV isolation, optimizing and standardizing detection and analytical methods pose additional challenges for the clinical translation of EV-based biomarkers. For example, different EV detection technologies may enrich distinct EV subpopulations and show variable analytical sensitivity, leading to differences in EV quantification and molecular cargo profiling across studies. Among EV detection technologies, nanoflow cytometry enables single-particle analysis and phenotypic profiling of EV subpopulations, whereas surface plasmon resonance offers highly sensitive, label-free detection. Microfluidic platforms have attracted considerable interest because they integrate EV isolation and analysis while requiring minimal sample volumes. More recently, ultrasensitive approaches such as single-EV analysis and thermophoretic assays have demonstrated the potential to detect rare disease-associated vesicles at very low concentrations. However, despite their promising analytical performance, most of these technologies still face challenges related to high costs, technical complexity, limited standardization, and insufficient large-scale clinical validation, which currently hinder their widespread clinical implementation [179].

EV features are also highly influenced by pre-analytical and analytical variables, including sample collection procedures, the choice of biological matrix (plasma versus serum), anticoagulant type, and sample processing protocols. Biological factors such as physical activity, lifestyle habits, and other individual physiological conditions may also influence circulating EV release and cargo composition [101]. Moreover, identifying potentially disease-associated EV cargo must be followed by rigorous analytical and clinical validation, ideally in independent and sufficiently representative patient cohorts, to establish its reliability as a biomarker [180]. Therefore, standardized workflows that integrate EV isolation, detection, molecular cargo analysis, and biomarker validation are essential to translate EV-based biomarkers into clinical practice reliably [99].

Alongside direct analysis of patient-derived biological fluids, in vitro cell culture models provide a complementary preclinical platform for discovering and characterizing EV-associated biomarkers under controlled, reproducible conditions. Indeed, in vitro models enable rapid screening and straightforward investigation of potential biomarker candidates before assessing them in complex biofluids. Moreover, this approach is particularly relevant in tumors and other pathologies, where circulating EVs are heterogeneous and disease-associated signals may be difficult to distinguish from EVs released by sources other than cancer cells (e.g., platelet-derived EVs). Accordingly, EVs released by cultured tumor cells can serve as a more manageable source for identifying candidate molecules for later investigation in biofluids.

However, the translational relevance of this approach depends critically on the biological model used for EV analysis. As discussed above, differences in cellular phenotype, culture conditions, tissue architecture, and microenvironment can substantially affect EV secretion, abundance, molecular cargo, and functionality [86]. Consequently, EVs derived from different in vitro models may display distinct molecular profiles, potentially influencing the evaluation and identification of potential biomarker candidates. Therefore, it is needed to carefully consider the experimental model when using cell-derived EVs as a preclinical tool for biomarker discovery, as an inappropriate or insufficiently representative model may yield molecular signatures that do not adequately reflect those detected in vivo.

Several studies have demonstrated that EVs isolated from in vitro models share molecular features with EVs collected from patient-derived biofluids. In some cases, EV-associated molecular signatures identified in conventional 2D culture systems have also been detected in patient plasma-derived EVs, supporting the use of these models for the discovery of potential candidate biomarkers. For example, EVs released by hypoxia-exposed colorectal cancer (CRC) cells have been used to identify hypoxia-associated miRNAs, including miR-486-5p, miR-181a-5p, and miR-30d-5p, which were subsequently assessed in plasma-derived EVs from patients with locally advanced CRC [181]. Similarly, EVs derived from CRC cell lines display molecular signatures associated with specific tumor subtypes that are also detectable in the patient’s plasma-derived EVs [182]. These observations support using conventional 2D cultures for preclinical studies.

However, because EVs in patient biofluids originate from multiple cellular sources, shared molecular features between cell-derived and patient-derived EVs should not be interpreted as evidence of biomarker detectability, disease specificity, or clinical applicability. Instead, they support identifying candidate biomarkers that require subsequent analytical and clinical validation to demonstrate clinical utility.

Comparative studies, however, suggest that EV profiles are influenced by model complexity. For example, EVs derived from cervical cancer cells cultured in 3D showed greater concordance with patient plasma EV small RNA profiles than EVs derived from 2D cultures. The reported ~96% similarity reflected overlap in small RNA profiles and should therefore be interpreted as molecular concordance rather than evidence of biomarker detectability, specificity, or clinical performance. In addition, several miRNAs identified in patient-derived EVs were detected in 3D-derived EVs but not in 2D-derived EVs, suggesting that 3D models may better recapitulate specific in vivo EV-associated molecular features. Similarly, studies using patient-derived PDAC organoids [183], and 3D ovarian cancer models [184] have identified EV-associated miRNA and protein signatures that overlap with those detected in patient plasma, supporting the potential value of advanced culture models for biomarker discovery.

**Figure 5:**
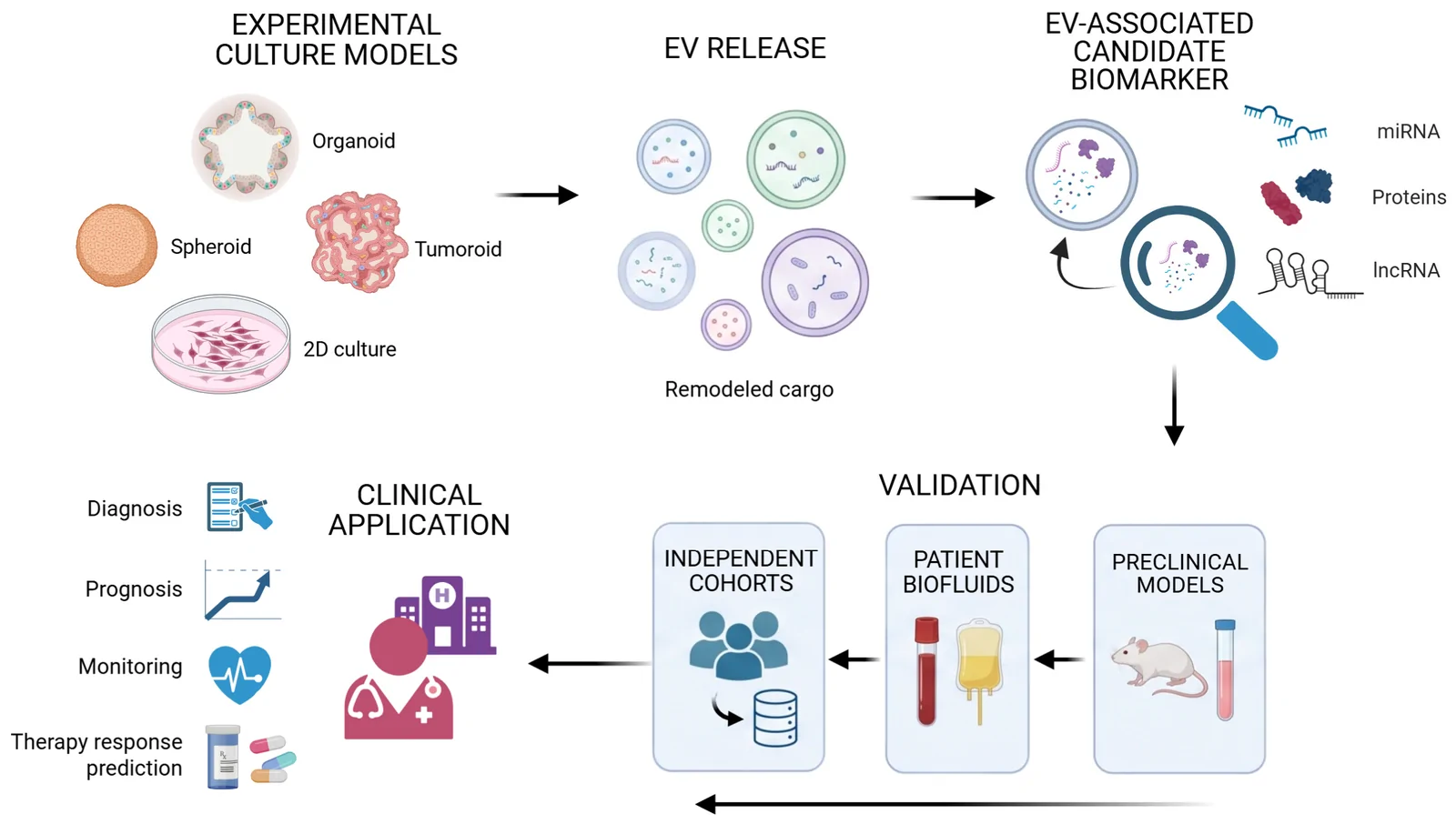
From Culture Models to Clinical Use: The Journey of Extracellular Vesicle Biomarkers. Experimental culture models influence EV features and associated molecular signatures. These signatures provide candidate biomarkers that can be identified and subsequently validated in preclinical models and patient-derived biofluids before potential clinical application. Created in BioRender. Poppa, G. (2026) https://BioRender.com/lymbhrg.

Nevertheless, studies systematically comparing EV signatures generated in 2D or 3D culture systems with those detected in patient biofluids remain limited. Therefore, current evidence primarily supports using advanced culture models to generate hypotheses and discover candidate biomarkers. Whether these candidates can ultimately become clinically useful biomarkers remains to be established through rigorous analytical and clinical validation, as well as demonstration of clinical utility. Bridging this gap will be essential to define the translational value of culture-derived EV signatures and to exploit EVs as clinically applicable biomarkers fully. Collectively, these observations support a translational framework in which culture model selection influences cellular phenotype, EV release and cargo composition, affecting the identification of clinically relevant EV-associated biomarkers (Figure 5).

## 6. Conclusions

EVs have emerged as promising sources of diagnostic, prognostic, and predictive biomarkers because they reflect the molecular features of their parent cells and are accessible through minimally invasive liquid biopsy approaches. However, the successful translation of EV-based biomarkers into clinical practice requires not only robust analytical and isolation procedures but also biologically relevant preclinical models for biomarker discovery and validation. Growing evidence indicates that the predictive value of EV-associated biomarkers depends not only on their molecular cargo but also on the experimental model used to identify them. While conventional 2D cultures remain valuable tools for the initial screening of candidate biomarkers, 3D and patient-derived models may, in specific experimental contexts, more closely reproduce EV molecular signatures observed in vivo or in clinical samples.

Current evidence suggests that increasing biological complexity, from conventional 2D cultures to advanced 3D systems, can provide additional information on clinically relevant EV-associated molecular signatures, highlighting the influence of model-dependent variables on biomarker discovery.

However, as discussed above, greater biological complexity does not necessarily confer greater predictive or translational value, and no single experimental model can be considered universally optimal. Instead, it should be considered to select the most appropriate model based on the specific research question, biological context, and intended application. Further comparative studies directly integrating 2D and 3D models with patient-derived biofluids are needed to determine which models most reliably reproduce clinically relevant EV signatures. Future efforts should also prioritize standardized EV isolation and characterization procedures, and validation in independent patient cohorts, while establishing clearer criteria for selecting the most appropriate preclinical model based on the specific research question and intended biomarker application.

## Figures and Tables

**Table 1:** Main molecular components of extracellular vesicles and their biological functions.

Component	Example	Function	Ref.
Lipids	Sphingolipids (sphingomyelin, ceramide); glycolipids (gangliosides); glycerophospholipids (phosphatidylcholine, phosphatidylinositol); glycerolipids (diacylglycerol); sterols (cholesterol); bioactive lipids (arachidonic acid, prostaglandin E2)	Structural role in the membrane; Modulation of recipient cell state; Influence EV properties and mechanisms of action.	[24,25,26,27,28,29]
Proteins	Lipid metabolism enzymes (phospholipases D and A2), ESCRT components, Tetraspanins (CD9, CD63, CD81, CD82), galectins, and integrins, proteins involved in EV biogenesis and release (RAB27A, RAB11B, ARF6), signal transduction proteins (EGFR), antigen-presenting proteins (MHC class I and II), transmembrane proteins (LAMP1, TfR), GTPases, annexins, flotillins, ALIX, and TSG101	Involved in the mechanisms responsible for EV biogenesis; Enable EV interaction with membrane proteins of target cells through ligand–receptor systems; Promote internalization and the release of their bioactive cargo.	[24,30,31]
Nucleic acids	Genomic DNA, mitochondrial DNA, mRNA, and many types of non-coding RNAs, including miRNA, long non-coding RNAs, circular RNAs, small nucleolar RNAs, small nuclear RNAs, tRNAs, and rRNAs.	DNA cargo may contribute to intercellular genetic information transfer. RNA cargo can be delivered to recipient cells, contributing to intercellular communication.	[6,21,32,33]

**Table 3:** EV molecules with biomarker potential in neoplastic and non-neoplastic disease.

Disease	EV-Associated Marker	Biofluid	Potential Biomarker Type	Ref.
***Neoplastic disease***				
Nervous system tumors				
Neuroblastoma	Hsa- miR-199a-3p	Blood plasma	Diagnostic/prognostic	[114]
Neuroblastoma with bone marrow metastasis	miR-375	Blood plasma	Prognostic/disease-status biomarker	[115]
Glioblastoma	miR-21	Cerebrospinal Fluid	Diagnostic	[116]
	17K-uEV protein	Urine	Diagnostic, monitoring, response	[112]
Gastrointestinal tumors				
Hepatocellular carcinoma	miR-18a, miR-221, miR-222, miR-224 miR-101, miR-106b, miR-122, miR-195	Blood serum	Diagnostic	[117]
	miR-665	Blood serum	Diagnostic/prognostic	[118]
Pancreatic cancer	GPC1	Blood serum	Diagnostic/prognostic	[119]
	WASF2 mRNA, ARF6 mRNA, SNORA74A, SNORA25	Blood serum	Diagnostic	[120]
Pancreatic ductal adenocarcinoma	miR-10b, miR-21, miR-30c, miR-181a, miR-let7a	Blood plasma	Diagnostic/differential diagnosis (vs chronic pancreatitis)	[121]
Liver cancer	AnnV, CD113, gp38, EpCAM panel	Blood serum	Diagnostic	[113]
Respiratory tract cancers				
Lung adenocarcinoma	miR-200b/c, miR-141, miR-375, LCN2 mRNA	Pleural effusion	Diagnostic	[122]
	miR-21-5p, miR-126-3p, miR-140-5p	Blood serum	Early diagnosis	[123]
Non-small cell lung cancer	PD-L1	Blood plasma	Predictive/prognostic	[111]
Female tumors				
Breast cancer	MMP-1/CD63, miR-21	Urine	Early diagnosis	[124]
	EpCAM, HER2	Blood plasma	Diagnostic	[125]
Invasive ductal cancer	miRNA-223-3p	Blood plasma	Differential diagnosis (vs ductal carcinoma in situ)/early detection of invasive disease	[126]
Ovarian cancer	miR-200c, miR-145, miR-93	Blood serum	Diagnostic	[127]
	PLEK1, TRANK1, BLTP1, PCDHGC5, SERPINA10 PTGDS, IGKV1-27, PON3, CFD, C1RL	Blood serum Ascites	Diagnostic/prognostic	[109]
	miRNA-205	Blood plasma	Diagnostic, early detection/disease severity/staging-associated	[128]
Uro-genital tumors				
Prostate cancer	GDF15, SLURP2, SLIT1, AKAP9	Urine	Differential diagnosis (vs prostatitis)/diagnostic	[110]
	PSA/CD81	Blood plasma	Diagnostic	[129]
Bladder cancer	UCA1-201, UCA1-203, MALAT1, LINC00355	Urine	Diagnostic	[130]
Renal carcinoma	miR-210, miR-1233	Blood serum	Diagnostic	[131]
Other tumors				
Melanoma	PDCD6IP (ALIX)	Blood plasma	Prognostic	[132]
	miRNA-532-5p, miRNA-106b	Blood serum	Diagnostic/monitoring	[133]
Acute myeloid leukemia	miR-10b	Blood serum	Prognostic	[134]
Thyroid carcinoma	miR-5189-3p	Blood plasma	Diagnostic	[135]
Papillary thyroid carcinoma	miR-346, miR-10a-5p, miR-34a-5p	Blood plasma	Diagnostic	[136]
	miR-485-3p, miR-4433a-5p	Blood plasma	Diagnostic	[137]
***Non-neoplastic-disease***				
Neurological disorders				
Parkinson’s disease	α-synuclein	Blood plasma and serum	Differential diagnosis (vs multiple system atrophy)	[138]
Gastrointestinal diseases				
Non-Alcoholic Fatty Liver Disease	FZD-7	Blood plasma	Diagnostic/prognostic	[139]
Hepatic fibrosis in chronic hepatitis B	miR-92a-3p, miR-146a-5p	Blood serum	Diagnostic	[140]
Liver fibrosis	miR-122	Blood serum	Diagnostic	[141]
Respiratory tract disorders				
Idiopathic pulmonary fibrosis	miR-142-3p, miR-33a-5p, let-7d-5p	Sputum	Diagnostic/disease severity-associated	[142]
Cardiovascular disorders				
Myocardial infarction	Protein panel (ApoC-III, ApoD, GP Ibα, C1qA, C5)	Blood plasma	Diagnostic	[143]
	miR-126	Blood plasma	Prognostic	[144]
Coronary artery disease	miR-382-3p, miR-432-5p, miR-200a-3p, miR-3613-3p	Blood plasma	Diagnostic	[145]
Acute coronary syndrome	miR-208a	Blood serum	Early diagnosis/prognostic	[146]
Heart failure	miR-22, miR-320a, miR-423-5p, miR-92b	Blood serum	Diagnostic/prognostic	[147]
	miR-92b-5p	Blood serum	Diagnostic	[148]
	miR-34a, miR-194	Blood serum	Predictive	[149]
	miR-425, miR-744	Blood plasma	Predictive	[150]
Urogenital disorders				
Diabetic nephropathy	Aquaporins AQP2, AQP5	Urine	Diagnostic/monitoring	[151]
Lupus nephritis	miR-146a	Urine	Diagnostic/monitoring	[152]

**Table 4:** Main advantages and disadvantages of the principal EV isolation methods.

Isolation Method	Separation Principle	Advantages	Disadvantages	**Ref.**
Ultracentrifugation	Sedimentation	- Simple and adaptable setup - Availability of established protocols - Ability to process large sample volumes	- Requirement for specialized equipment - Lack of unified protocols - Time-consuming workflow - Low purity due to co-pelleting of contaminants - Low throughput - Potential impairment of EV integrity/structure	[161,167,168,169,170,171]
Size-Exclusion Chromatography (SEC)	Particle size	- Ease of use - Rapid processing - Preservation of EV integrity/structure - High purity - High yield - High reproducibility	- Requirement for protocol optimization - Potential contamination from co-eluting non-EV particles - Limited sample volume capacity per run - High cost - Low throughput	[167,168,169,170,172,173]
Polymer Precipitation	EV–polymer interaction	- Ease of use - No requirement for specialized equipment - Rapid processing - High yield - Preservation of EV integrity/structure - Potential for automation	- Risk of co-isolating non-vesicular contaminants and polymer residues - Poor reproducibility	[167,168,170]
Immunoaffinity Capture	Surface markers	- High specificity and purity - Suitable for small sample volumes - Potential for automation	- Time-consuming workflow - High cost of reagents - Limited scalability for large volumes - Potential failure to isolate the full heterogeneous EV population - Risk of non-specific binding - Potential EV damage from eluting buffers	[167,168,169,170,174]
Microfluidics	Surface markers, size, density	- Rapid processing - Suitable for small sample volumes - High purity and recovery - Potential for automation	- Requirement for specialized equipment - Lack of uniform protocols - Limited capacity for large sample volumes - Susceptibility to clogging	[166,168,169,170,175,176,177]

Sedimentation depends on shape, size, density, sample viscosity; purity is intended as the ratio of EVs to total protein; the lack of integrity implies a possible loss of biomolecular content.

## Data Availability

No new data were created or analyzed in this study. Data sharing is not applicable to this article.

## Abbreviations

The following abbreviations are used in this manuscript:

2D	Two-dimensional models
3D	Three-dimensional models
ABs	Apoptotic Bodies
CEA	Carcinoembryonic antigen
circ-RNA	circular-RNA
CRC	Colorectal cancer
DC	Differential centrifugation
ECM	Extracellular Matrix
EPI	ExoDx Prostate IntelliScore
EVs	Extracellular Vesicles
EXOs	Exosomes
lEVs	Large EVs
ILVs	Intraluminal Vesicle
ISEV	International Society for Extracellular Vesicles
lncRNA	long non coding-RNA
miRNA	microRNA
ncRNAs	microRNA
MVBs	Multivesicular bodies
MVs	Microvesicles
NTA	Nanoparticle Tracking Analysis
PSA	Prostate-specific antigen
SEC	Size Exclusion Chromatography
sEVs	Small EVs
SP-IRIS	Single particle interferometric reflectance imaging sensing
TME	Tumor microenvironment
UC	Ultracentrifugation
